# P-wave duration and dispersion in patients with peripheral edema and its amelioration

**Published:** 2007-01-01

**Authors:** John E Madias

**Affiliations:** Mount Sinai School of Medicine, of the New York University, New York, NY and the Division of Cardiology, Elmhurst Hospital Center, Elmhurst NY , USA.

**Keywords:** Electrocardiology, electrophysiology, P-wave duration, P-wave dispersion, low voltage ECG, peripheral edema, hemodialysis, congestive heart failure

## Abstract

**Background:**

Attenuation of the P-wave amplitudes in patients with peripheral edema (PERED) has been recently reported, with P-waves regaining some of their amplitude in patients, who subsequently experienced amelioration of their PERED. Changes in the P-waves correlated with the corresponding alterations in the QRS complexes. Also since amplitudes and durations of QRS complexes changed in parallel in patients with PERED, it was hypothesized that similar changes in the P-wave amplitudes, mean P-wave duration (P-du-mean), and P-wave dispersion (P-d), would occur in such patients.

**Methods:**

Measurements of P-wave amplitude, P-du-mean and P-d in patients who developed, or experienced alleviation, of PERED, were carried out and analyzed.

**Results:**

Although P-wave amplitudes and P-wave areas decreased with development of PERED (N = 16), and increased with its amelioration (N = 6), P-dur-mean before PERED was 66.8±14.5 ms, and at peak weight gain it was 65.2±11.9 ms, p = 0.66; also at peak weight gain and subsequent lowest weight, in the patients who lost weight, it was 66.5±9.9 ms and 72.3±12.0 ms, respectively, p = 0.38. Similarly the P-d prior to PERED was 62.3±25.2 ms, and at peak weight gain it was 74.3±29.3 ms, p = 0.09; also at peak weight and subsequent lowest weight, in the patients who lost weight, it was 58.8±34.2 ms, and 61.3±13.6 ms, respectively, p = 0.87.

**Conclusion:**

P-du-mean and P-d did not change in patients who developed PERED; their stability is attributed to the offsetting of the electrophysiologically-mediated *real* changes, by opposite *apparent* changes, imparted by PERED.

## Introduction

Attenuation of the amplitude of P-waves in patients developing peripheral edema (PERED), similar changes in the respective QRS complexes, and increase in weight, with good inter-correlations, have been previously reported [1]. Since duration of QRS complexes changed in parallel with amplitude of QRS complexes in the same patients [2], it was hypothesized that similar changes in the P-wave duration, P-wave dispersion (P-d), and amplitude of P-waves would occur in such patients with PERED. Previously, P-waves were measured manually and reported as sums of the amplitude of all 12 ECG leads in mm (ΣP) [1]. Automated measurements [3] on the same ECGs of an array of additional P-wave variables, not included in the first report [1],  e.g., mean P-wave duration (P-du-mean), and P-d, were used in the present study. The last 2 have been investigated intensely in the past, and increased values have been found in patients with cardiac and other pathology [4-23]. Importantly, increased P-du-mean and P-d have been shown to be predictive of atrial fibrillation (AF) in general, long term vs. short term AF, and AF after cardiothoracic surgery, mostly coronary bypass grafting [4-6,8,11,13,15-23]. Increase in P-du-mean is felt to be reflective of intra-atrial conduction delay, and thus it signifies an *electropysiological* disturbance. Measurements of P-du-mean in all above studies were carried out manually (employing all leads or selective ones, e.g., lead II), or via automated computer algorithms. Also P duration assessed by signal averaged ECG has been implemented successfully for the prediction of post-operative AF [24-27]. Moreover decrease in the P-du-mean has been documented in patients with congestive heart failure (CHF), responding to diuresis [28] or after nitroprusside infusion [29], and increase in patients undergoing hemodialysis (HD) [30,31]. It is intriguing that alleviation of fluid overload in patients with CHF and after HD [28,30,31] led to *divergent* effects on the P-du-mean. The objective of the present study was to evaluate several P-wave variables (mainly P-du-mean and P-d) in patients with PERED, and its subsequent partial alleviation.

## Material and methods

### Study patients

The 28 patients with and 28 "controls" without PERED, employed in a previous report [1], were considered herein; only 16 of the PERED patients with sinus rhythm on admission and at peak weight gain (47.5±29.6 lbs, 32.6±22.2%) were studied; 12 patients underwent pacemaking, or suffered AF. Six patients with PERED, who lost subsequently weight (48.8±26.6 lbs, 21.5±11.4%), were re-studied. The PERED patients presented with hypertension, pneumonia, exacerbated chronic obstructive lung disease, respiratory arrest, respiratory failure, acute respiratory distress syndrome, sepsis, anoxic encephalopathy, or a combination of ≥2 of the above, in addition to their chronic conditions like diabetes mellitus, coronary artery disease, stroke, tuberculosis, and other miscellaneous, renal, and gastrointestinal afflictions [32].

From the 28 "controls", 20 were included, since 8 had pacemaking/AF, and presented with various cardiovascular and other illnesses (peumonia, respiratory arrest, respiratory failure, chronic obstructive lung disease, CHF, or a combination of ≥2 of the above), requiring admission [32], and had ECG measurements on admission and at discharge.

### Study variables and measurements

**Figure 1:**
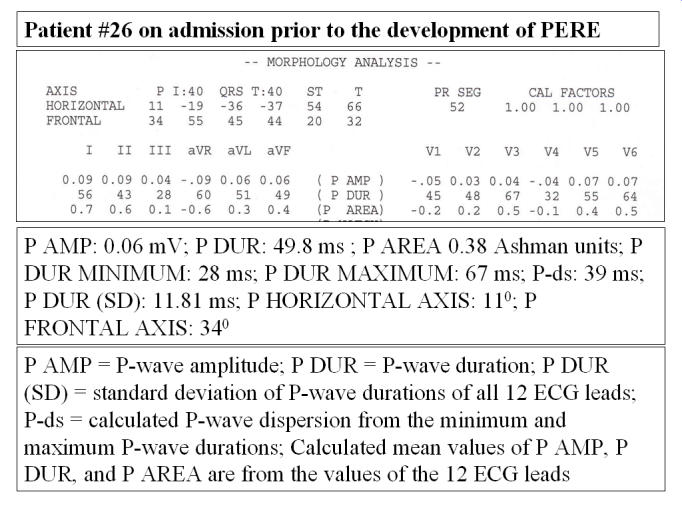
Reproduction of the pertinent portion of the "Extended measurement Report" provided by the automated measurement program [3], with the values of measurements for all 12- ECG leads, and the calculated variables based on such measurements.

**Figure 2:**
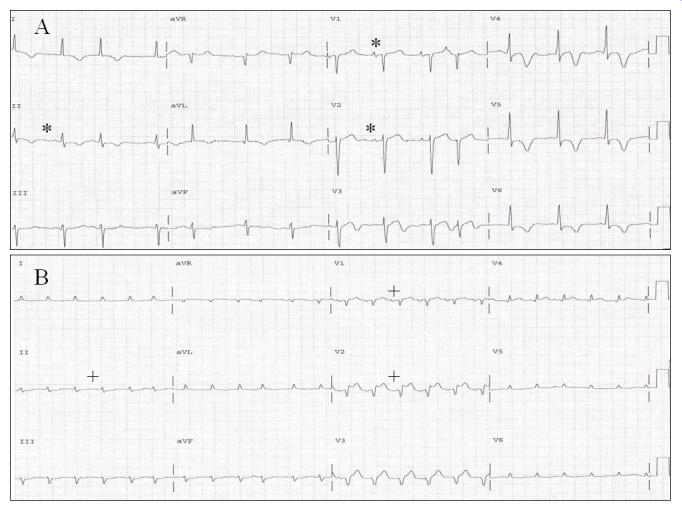
Standard ECGs of patient #13 on admission (A), and after he gained 41.6 lbs (18 9 Kg) (28.9 %) in the process of developing PERED; P-waves in leads II, V1, and V2 before PERED (*), and after PERED (+).

Details on patients and study design can be found elsewhere [32];  variables used herein included the P-du-mean, maximum P-du (P-max), minimum P-du (P-min), the standard deviation of the P-du from all 12 ECG leads (P-du-SD) [33], all in ms, the mean P-wave area in "Ashman" units (P-area-mean) (1 Ashman unit = an area of 1.0 mm^2^) [3,34], the P-d (P-max  -  P-min, using all 12 ECG leads) in ms, the mean P-amp in mm (P-amp-mean), and the horizontal axis of the P-waves (P-horiz-axis) in degrees. Data on the P-wave frontal axis (P-fro-axis), ΣP in mm, P-R intervals, and heart rates has been reported previously [1]. The P-du-mean and mean P-area were calculated by summing the individual such P-wave values from all 12 ECG leads, provided by the automated program (HP, now Philips M1700A PageWriter model) [3]Figure 1) and dividing by 12. This automated measurement and interpretation program has been previously validated [35,36]. The software could measure P-wave duration in all 12 leads in 31 of 38 instances (16 from admission, 16 from the time of peak weight, and 6 from the time of lowest weight) (81.6%), which represented all 16 patients on admission, 9 of the 16 patients at the time of the peak weight, and all 6 patients, at the time of subsequent, to the time of the peak lowest weight, for the patients who lost weight. In the other 7 patients the software could measure only 10 leads (excluding V5 and V6) in patient #1, 11 leads (excluding III) in patients #4 and #13, 11 leads (excluding I) in patient #5, 7 leads (excluding aVL and V3-V6) in patient #11, 8 leads (excluding aVL and V4-V6) in patient #23, and 7 leads (excluding I, aVL and V4-V6) in patient #26. For calculation of the P-du-mean in instances with measurements of fewer than 12 leads the sums of the values of the P-wave durations, were divided by the number of the leads with successful measurement. Values of ΣP [1] (manual measurements) were used for comparisons with the mean P-wave amplitude (P-amp-mean), which was calculated as the sum of the values of all ECG leads, provided by the automated ECG program, divided by 12. For all above measurements data were obtained or calculated by taking in consideration the printed form of the "Extended Measurement Report" generated by the automated program [3], part of which is reproduced in the upper panel of Figure 1. Data acquisition was the same for all patients at baseline, the time of the peak weight gain, and the time of subsequent lowest weight (for patients who lost weight). The automated system does not generate records of the ECG measurements, with caliper markings indicating the onset and offset of P-waves, used in the measurement of P-wave duration, but only provides the numerical value of the measurements. The automated measurements of the P-wave are part of the overall ECG measurements of the HP ECG Analysis Program, and it is based on simultaneous 12-lead acquisition of the ECG. Calibration of the ECG recordings was 10 mm = 1.0 mV, and the paper speed was 25 mm/sec. Automated measurements of the variables provided inclusion of unbiased data for analysis. Figure 2 illustrates the minuscule amplitude and duration of P-waves particularly with PERED, which makes it impossible to measure manually, and explains the necessity of using automation in acquisition of parameters displayed in Figure 1. Serum K^+^, Ca^++^, Mg^++^, and HCO3 were monitored during the study.

### Statistical analysis

Continuous data are presented as mean±SD, and were analyzed with the unpaired and paired t tests, as indicated. The Levene test [37] was used in conjunction with the unpaired t test to decide whether the population variances for the subgroups of patients with PERED and the "controls" were equal or not, and thus the "pooled variance estimate" or the "separate variance estimate" should be employed respectively for the assessment of the significance level. Nominal data were analyzed by the chi square test, and associations between continuous data were evaluated by Pearson correlation analysis. All statistical operations were 2-tailed [38].  The SPSS/PC+ 4.0.1 statistical package [36] was used, and a p<0.05 was taken as statistically significant.

## Results

### Patients with PERED vs. "controls"

No difference in age, sex distribution, ΣP, P-fro-axis, P-horiz-axis (p = 0. 56), P-amp-mean (p = 0.18), P-area-mean (p = 0.054), P-d (p = 0.4), P-max (p = 0.051), and P-du-SD (p = 013), were noted in the patients with PERE and the "controls" on admission [1];  however heart rates were faster (p = 0.002), and P-R intervals were shorter (p = 0.007), P-du-mean, (p=0.003), and P-min (p = 0.047) were shorter in patients with PERED (Table 1 and 2). Electrolytes in patients with PERED were unchanged.

### Manual vs. automated measurements in patients with PERED

Correlation of automated P-amp-mean and manual ΣP was significant (r = 62, p =0.01). Comparing the manual and automated measurements of the amplitude of P-waves (multiplying the latter by 12) on Table 1, it appears that manual measurements underestimated the P-wave amplitude at the time of peak weight gain, for the 16 patients with PERED and the 6 patients who lost weight, and overestimated it at the time of the lowest weight. P-amp-mean decreased at the time of peak weight and increased at the time of weight loss (Table 1), as noted previously with manual measurements (ΣP) [1].

### Manual vs. automated measurements in "controls"

Correlation of the automated P-amp-mean and the manual ΣP [1] in the "controls" was excellent on admission (r = 0.91, p = 0.00005), and at discharge (r = 0.94, p = 0.00005). Comparison of the values of ΣP to the P-amp-mean (multiplying the latter by 12) on Table 2 reveals the agreement of the amplitudes of P-waves derived by the 2 methods.

### Correlations of patients with PERED

Correlation of the change in the automated P-amp-mean and peak weight gain (r = 0.80, p =0.0002) for the patients with PERED was good; however such correlations were poor for the patients who lost weight, at their peak weight (r = 0.55, p = 0.26), and subsequent lowest weight (r = 0.75, p = 0.09) (Table 1). P-area-mean at baseline, peak weight, and lowest weight followed the same pattern as the P-amp-mean (Table 1). On the other hand the 4 parameters reflecting P-duration, the P-d, and the P-horiz-axis did not change with the fluid perturbations (Table 1). The behavior of P-horiz-axis corroborated the previous findings on P-fro-axis [1].

### Comparisons in "controls"

All P-wave and other parameters in the 20 "controls" did not change between admission and discharge, save for the P-area-mean, which decreased at discharge in comparison with admission (Table 2).

## Discussion

Changes of P-du-mean and P-d have been observed in patients with PERED (e.g., treated for CHF or after HD) [28-31], which suggest that PERED should be taken into account, when employing P-wave indices. Management of CHF by diuresis led to reduction of P-du-mean and P-max, with an inverse correlation between P-du-mean and fluid volume lost (r = -0.59, p = 0.015) [28], viewed as amelioration of electrophysiology disturbances, caused by volume overload. Also, signal averaged P-du, was longer in patients with higher pulmonary capillary wedge pressure, and reduction of the latter by nitroprusside, led to reduction of the P-du, although left atrial diameter did not correlate with P-du [29]. In contrast, alleviation of fluid overload by HD resulted in lengthening of P-max and P-d (considered arrhythmogenic) [30,31], and attributed to electrolyte imbalances, or the HD itself. Changes in the P-waves were more notable in patients with left atrial diameter >45 mm [30]; also P-max and P-d correlated with biochemical parameters, effected by HD [31]. The above P-wave changes are presumed to be *electrophysiologically-mediated*, reflective of alterations imparted on the heart, and thought to have clinical consequences.

It is paradoxical that improvement of the fluid overload in patients with CHF and after HD produced *divergent* responses in the P-du and P-d. Furthermore, the present report showed *no changes* in P-du-mean and P-d in patients with a variety of critical illnesses and marked PERED (Table 1). The reliability of this observation was strengthened by the stability of the P-du-mean and P-d in patients with PERED who lost weight, (Table 1), and in the "controls", who had stable weights during hospital stay (Table 2). P-wave indices were unchanged throughout this study, in-spite of major perturbations of the degree of PERED of patients; also values of P-wave parameters of PERED patients and the "controls" were similar to the ones described in previous studies [4-23]; P-max and P-d of PERED patients and the "controls" were similar to the ones reported in patients with paroxysmal AF and large left atrial distensions, and higher than reported for subjects without history of cardiovascular illness [17,22]. Values of P-max were higher for patients with CHF than the "controls" without known heart disease in a previous study, but such measurements were made by signal-averaged techniques [29]. All the above suggest that the interplay of the P-du and P-d with PERED in patients with different illnesses may be complex. P-du-mean and P-d are impacted by changes in the preload and afterload [16,28,29], circadian variation [34], baseline left atrial dimension and its change, atrial stretch and its relief [29,39], age, the state of the autonomic nervous system [40], and concomitant medications [40,41], all with *electrophysiological* influence on the heart; however PERED and its partial reversal *per se*,  also may have an impact on P-wave indices. PERED due to CHF may be different from that associated with the multiple non-cardiac illnesses as included in the present study. One would expect atrial loading and P-wave parameters to be directly correlated with PERED in CHF, and thus induce electrophysiological derangements, but not so in pneumonia and other non-cardiac illnesses. At first glance the patients of the present study, which did not have CHF as an inclusion criterion, would not be expected to show correlation of P-wave changes with PERED. However these patients had history of hypertension, coronary artery disease, received large volumes of intravenous fluids, gained in turn enormous weights, developing "anasarca" PERED. In this respect the patients of this study should be considered as afflicted by *both* cardiac and non-cardiac illnesses. It is also possible that a more homogeneous group of patients might have shown different response to PERED, than shown in this patient cohort with diverse pathology.

When volume perturbations are implicated for the changes of P-wave indices in patients, it is meant implicitly or explicitly that the hemodynamic changes mediate electrophysiological alterations, expressed as prolongation in such indices [4-41]. However this report aims at partially implicating the PERED *per se*, as a contributing *non-electrophysiologic* mechanism of changes in P-wave parameters. In such context P-waves undergo *apparent* (in contrast to real, i.e., electrophysiologically-mediated) alterations due to the changes in the electrical properties of the "passive" volume conductor surrounding the heart. There is literature showing increase in the QRS complexes after HD [42-46], decrease and increase in the amplitude of QRS complexes and P-waves in patients with PERED and its amelioration [1,32],  and augmentation of QRS complexes with management of patients with CHF [47-49]. Commensurate with attenuation of the QRS complexes with PERED, reversible shortening of the QRS duration has been observed [2,50], which is not viewed as *real* (i.e., change in the ventricular conduction velocity and the duration of depolarization), but as *apparent*, resulting from failure of the measurement at the onset and offset of the QRS complexes, by which these portions of the QRS complexes are so low as to be indistinguishable from "noise", or are below the threshold of potential detection of the measuring algorithm [2]. By analogy one can envisage similar apparent changes in the P-du-mean and P-d, since the effect of the edematous body conductor is exerted proportionally on all components of the ECG curve, QRS complexes and P-waves alike [1,32]. An extreme precedent exists in the virtual *disappearance* of P-waves in patients with PERED where there are no detectable P-waves by either visual inspection, or by the automation a diagnosis of "junctional rhythm", is made, while intracardiac ECG reveals the presence of P-waves [51]. The effect of the gain/loss of excessive fluid in the "passive" body volume conductor has been discussed in detail elsewhere [1,32], and it leads to a decrease/increase in the "composite impedance" due to gain/loss of a constituent (water) with the *lowest* resistivity in the body [52], thus resulting in attenuation/augmentation of potentials of the entire PQRSTU ECG curve, and as per Ohm's law (V = I x R, where V = voltage, I = current, and R = resistance) [53-57].

**Figure 3:**
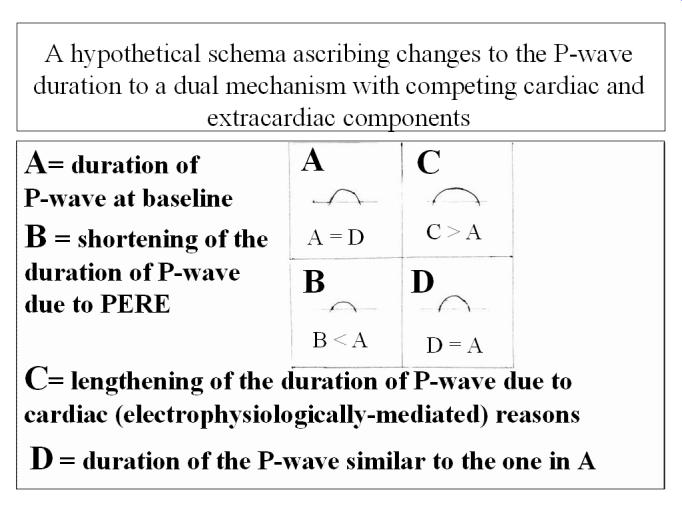
Hypothetical cardiac and extracardiac influences on the P-wave duration.

The above formulation does not imply that all the other known influences were not exerted in our patients with PERED. The edematous state produced reversible attenuation of the ΣP, P-amp-mean, and P-area-mean [1] (Table 1); however P-du-mean and all the other associated parameters did not change, as also noted in the "controls" who did not experience any changes in their body weights. These patients received large fluid volumes, suffered pneumonia and sepsis, were intubated, and developed acute renal failure [1,32], all conditions expecting to lead to prolongation of P-du-mean and P-d. Plausible explanations for the reduction of P-amp-mean, ΣP and P-area-mean, with unaltered P-durations are: 1) No changes in the P-durations occurred (Figure 3A), in-spite of the changes in P-wave amplitudes and areas. 2) Hemodynamic and or other electrophysiological influences leading to prolongation of the P-wave duration were exerted (Figure 3C), but they were *counteracted* by the shortening of the P-duration effect of PERED (Figure 3B), producing a cancellation effect (Figure 3D), and leading to an unchanged P-du-mean and P-d (Figure 3A and 3D). In short, it is proposed herein that the apparent influence of PERED has *cancelled out* the electrophysiologically-mediated real changes.

Measurements in this study were automation-based, and although they were used uniformly in all the patients with PERED and all the "controls", and for all 3 phases of the study, this constitutes a limitation of this work. However the automation system implemented has been previously repeatedly validated [35,36]. One should also keep in mind that particularly when the P-wave duration is the focus of an investigation, even manual measurements of magnified analog ECG tracings, or computer-based measurements with electronic calipers and operator input, are not devoid of difficulties, ambivalence, and uncertainty, as to where exactly is the onset and offset of P-waves.

## Figures and Tables

**Table 1:**
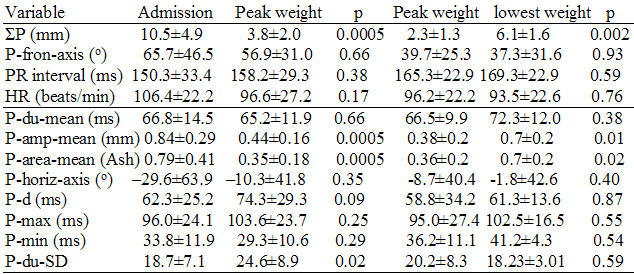
P-wave measurements on admission, peak weight, and subsequent lowest weight in patients developing PERED (N=16) and (N = 6).

Abbreviations as in the text. The values under the 2 headings of "Peak weight" are different, because the first reflect all the patients (N = 16) and the second reflect the sub-fraction of patients (N=6), who subsequently lost weight. Results above the line represent data published previously (with permission from the Madias JE. P-waves in patients with changing edematous states: Implications on interpreting repeat P-wave measurements in patients developing anasarca or undergoing hemodialysis. Pacing and Clin Electrophysiol 2004;27:749-56., Blackwell Publishing), and are included here to facilitate interpretation of the data of the present study.

**Table 2:**
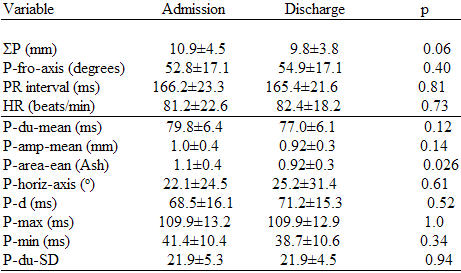
P-wave measurements on admission and discharge of "controls" (N=20)

Abbreviations as in the text. Results above the line represent data published previously (with permission from the Madias JE. P-waves in patients with changing edematous states: Implications on interpreting repeat P-wave measurements in patients developing anasarca or undergoing hemodialysis. Pacing and Clin Electrophysiol 2004;27:749-56., Blackwell Publishing), and are included here to facilitate interpretation of the data of the present study.
